# Helix-fixation leadless pacemaker delivery catheter reeling in intracardiac tissue and becoming stuck: A case report

**DOI:** 10.1016/j.hrcr.2024.04.010

**Published:** 2024-05-03

**Authors:** Takehiro Nomura, Daiki Kumazawa, Kosuke Onodera, Yosuke Mizuno, Shigeru Toyoda, Kennosuke Yamashita

**Affiliations:** ∗ Heart Rhythm Center, Department of Cardiovascular Medicine, Sendai Kosei Hospital, Miyagi, Japan; † Department of Cardiovascular Medicine, School of Medicine, Dokkyo Medical University, Shimotsuga-gun, Tochigi, Japan

**Keywords:** Leadless pacemaker, Complication, Tricuspid valve, Helix-fixation, Catheter stuck

**Key Teaching Points**

• Aveir (Abbott, Chicago, IL) leadless pacemakers and delivery catheters could snag intracardiac tissue when they are re-docked for retrieval or a replacement.
• If the delivery system is rotated with the snagged tissue, the catheter could reel in the tissue and become stuck.
• If the protective sleeves are not advanced smoothly, intracardiac tissue might become caught. In this situation, the Aveir system should be placed back in the tether mode and the position of the delivery catheter should be changed.

## Introduction

The Aveir (Abbott, Chicago, IL) is a helix-based active-fixation leadless pacemaker (LP) system. The complications associated with Aveir LP implantations include cardiac perforation, tamponade, stretched/broken helix, device dislodgement, premature battery depletion, and ventricular tachycardia.^1^^,^^2^ However, to the best of our knowledge, there have been no reports on the delivery catheter for the Aveir becoming snagged on and reeling in intracardiac tissue.

## Case report

**Figure 1:**
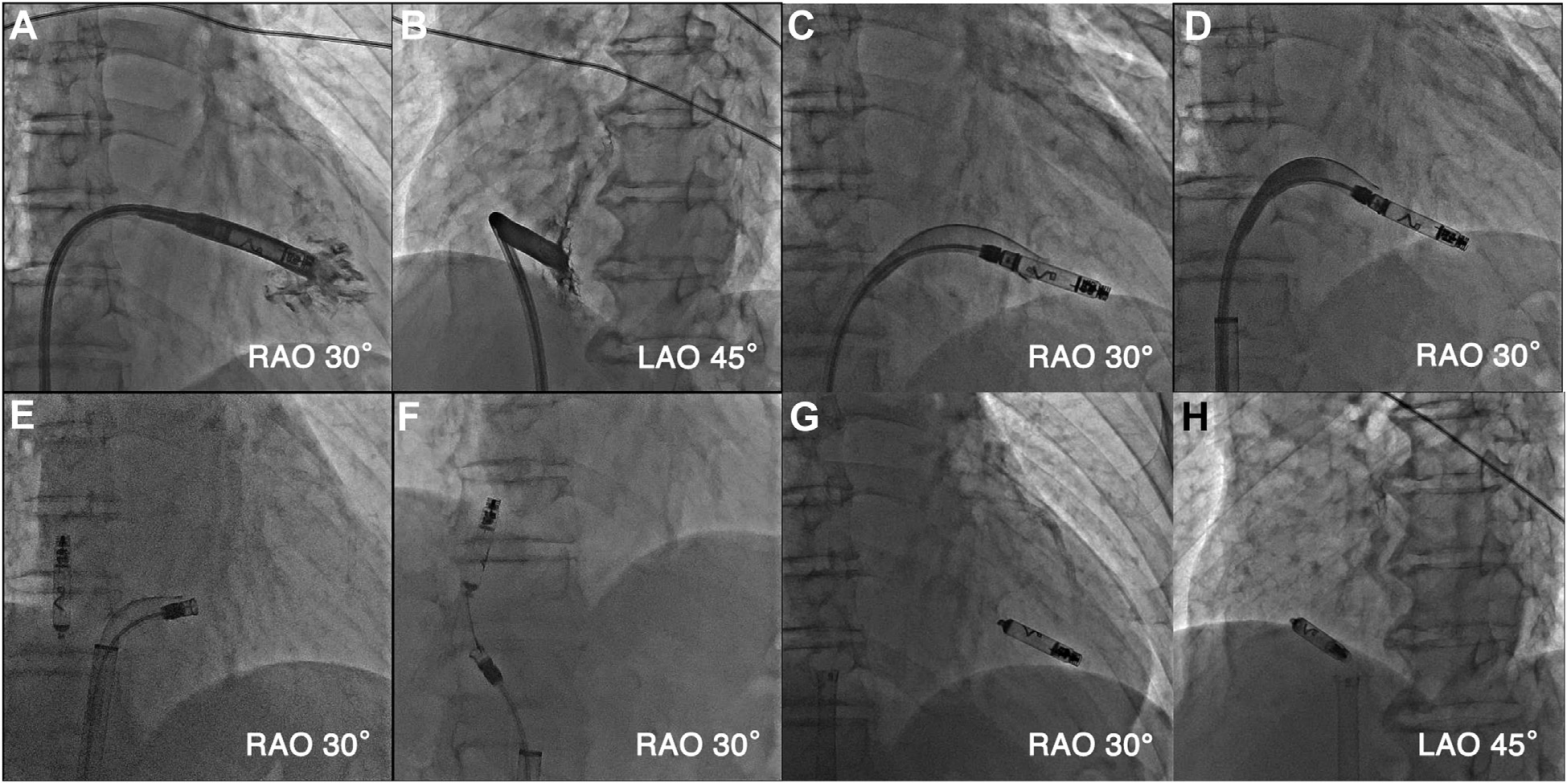
**A, B:** An Aveir VR leadless pacemaker (LP) (Abbott, Chicago, IL) was deployed onto the apical septum, but it was recaptured because of an unacceptable pacing threshold. **C:** The protective sleeve could not be advanced beyond the middle of the LP. **D:** The sleeve could no longer be advanced beyond the docking cap after counterclockwise rotation, and the catheter could not be retrieved. **E:** The catheter remained stuck, and the LP was dislodged after its release. **F:** The LP was successfully retrieved using an Aveir retrieval catheter. **G, H:** Another Aveir LP was successfully implanted after the LP that had migrated was retrieved.

**Figure 2:**
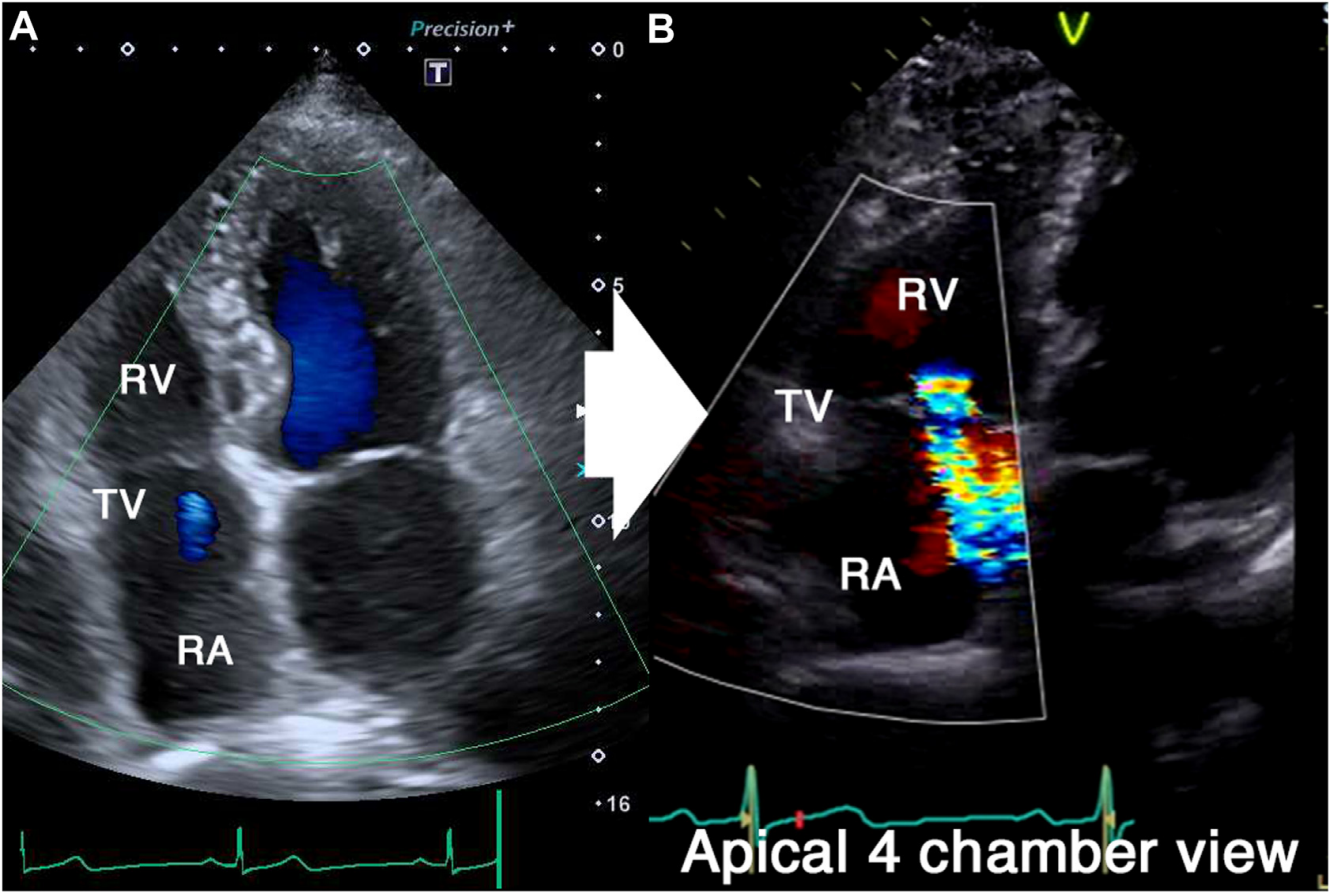
Transthoracic echocardiography before (**A**) and after the procedure (**B)**. The tricuspid regurgitation worsened from trivial (**A**) to moderate (**B**). RA = right atrium; RV = right ventricle; TV = tricuspid valve.

**Figure 3:**
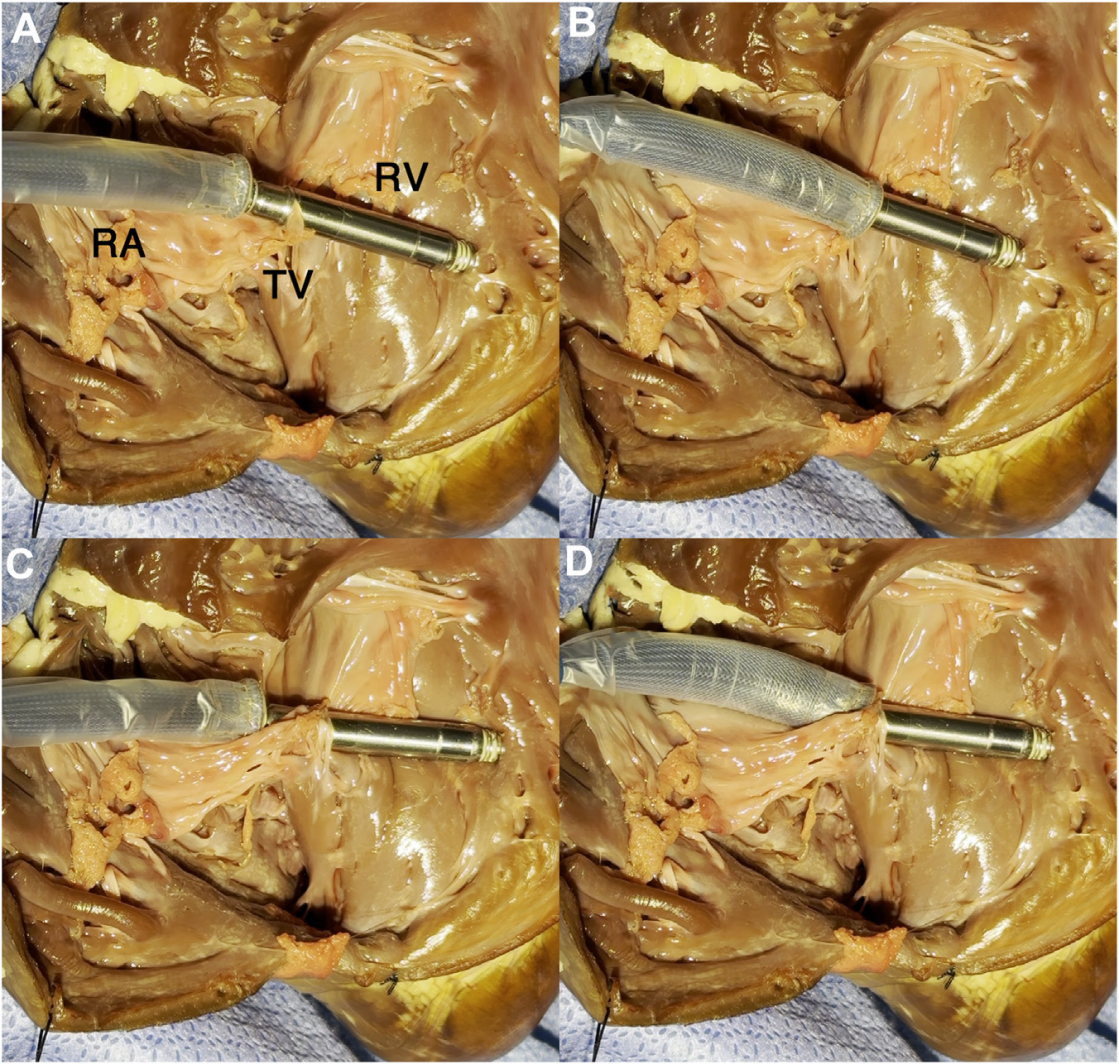
In vitro experiments were performed to replicate the Aveir (Abbott, Chicago, IL) delivery catheter becoming caught on and reeling in the porcine tricuspid valve (TV) leaflet. **A:** The porcine TV leaflet was tightly caught between the docking cap and leadless pacemaker. **B:** The protective sleeve could not be advanced beyond the middle of the device. **C:** The catheter began to reel in the leaflet with counterclockwise rotation; however, the pacemaker was successfully released from the myocardium. **D:** Compared to that before the counterclockwise rotation, the protective sleeve could be advanced even less.

A 74-year-old man was referred to our department for syncope. He had a history of an extensive encircling pulmonary vein isolation 3 years prior. His Holter electrocardiogram showed atrial fibrillation accompanied by sinus arrest for 5 seconds. The patient was diagnosed with syncope due to sick sinus syndrome, and the implantation of an Aveir VR LP (Abbott Medical, Chicago, IL) was scheduled. First, the LP was deployed onto the apical septum (Figure 1A and 1B). However, the pacing threshold was unacceptable even after waiting for 5 minutes; thus, the operator attempted to recapture the device and change its location. The protective sleeve did not advance beyond the middle of the device (Figure 1C and Supplemental Video 1). The rotational handle was rotated counterclockwise. However, although the chevron marker rotated, the system became stuck and could not be removed. Additionally, the protective sleeve retracted after it was rotated counterclockwise and it could no longer be advanced beyond the docking cap (Figure 1D and Supplemental Video 2). We assumed that the helix was fixed to the myocardium. Thus, we then released the LP and attempted to extract the delivery catheter. However, the LP migrated into the right atrium immediately after it was released. Contrarily, the delivery catheter became stuck around the tricuspid annulus. The delivery catheter, not the LP, was trapped and stuck in the intracardiac structures (Figure 1E). Hence, a surgical removal of the delivery catheter was considered, but the catheter was successfully removed when the handle was rotated clockwise (Supplemental Video 3). It was assumed that the tissue had coiled around the catheter by the counterclockwise rotation and was loosened by the clockwise rotation, allowing the catheter to be retrieved. The LP was retrieved using an Aveir retrieval catheter (Figure 1F). The catheter was oriented by the first operator, and the snare was operated by the assistant to catch the LP floating in the inferior vena cava. Another Aveir LP was successfully implanted after the LP that had migrated was retrieved (Figure 1G and 1H). No tissue was found adhering to the retrieved delivery catheter or LP. Figure 2A and 2B shows transthoracic echocardiography before and after the procedure, respectively. Tricuspid regurgitation worsened from trivial (Figure 2A) to moderate (Figure 2B). However, he had no symptoms of right heart failure and was discharged on postoperative day 4. We performed in vitro experiments to replicate this complication using a porcine heart purchased at a butcher shop. The porcine tricuspid valve leaflet became tightly caught between the docking cap and LP (Figure 3A and Supplemental Video 4). The protective sleeve could not be advanced beyond the middle of the pacemaker (Figure 3B). The catheter reeled in the leaflet with counterclockwise rotation; however, the pacemaker was successfully removed from the myocardium (Figure 3C). Compared to that before the counterclockwise rotation, the protective sleeve could be advanced even less (Figure 3D and Supplemental Video 5). However, a situation in which the delivery catheter remained stuck even after releasing the LP could not be replicated, and it was difficult to determine where the tricuspid valve tissue or chordae had become stuck on the catheter.

## Discussion

In the present case, the protective sleeve could not be advanced, and the tricuspid regurgitation worsened after the procedure, suggesting that the cardiac tissue around the tricuspid valve was caught when the LP and catheter were re-docked. The catheter most likely “reeled in” the tissue during the counterclockwise rotation and became tightly stuck. The protective sleeve retracting when it was rotated counterclockwise and then becoming free when it was rotated clockwise could also be explained using this mechanism. We were unable to distinguish whether the sheath became entangled with the tricuspid valve or its chordae tendineae. It was difficult to replicate the situation where only the catheter became stuck after the LP was released in the ex vivo experiment. However, by rotating the catheter in the opposite direction, the tissue that had become caught was successfully released without causing any significant damage. Considering the slight increase in the tricuspid regurgitation after the procedure, it should be reasonable to hypothesize that the catheter had become caught on either the tricuspid valve, its chordae, or both.

A previous report^3^ described a case of a tined-fixation LP that could not be retrieved because of tissue caught between the device and docking cap, similar to that in the present case. There was also a report of a flailing tricuspid leaflet and severe regurgitation after a tined-fixation LP implantation.^4^

When an LP is initially placed close to the base of the right ventricle, the tissue around the tricuspid valve may be more likely to become caught because the joint between the catheter and pacemaker is closer to the tricuspid valve. However, we believe that this complication could occur with all LP implantations. To prevent this complication from worsening, if the protective sleeve does not advance smoothly, the system should be placed in the tether mode, the angle of the delivery catheter should be changed, and a re-docking of the catheter and LP should be attempted. Moreover, if the system remains stuck after a sufficient counterclockwise rotation is performed, the delivery catheter, rather than the LP, may be trapped in the intracardiac structure. In the present case, the catheter was rotated clockwise to loosen the tissue adhesions and then could be removed. However, a clockwise rotation of the delivery catheter with an LP still docked may cause the helix to perforate the myocardium. Therefore, even if the delivery catheter is known to be stuck, the LP should be released first.

The use of transesophageal echocardiography or intracardiac echocardiography is deemed necessary to ascertain the reason for catheter entrapment, offering the potential to distinguish whether it involves the atrial muscle, chordae tendineae of the tricuspid valve, or tricuspid leaflets. However, those modalities may still not be sufficient to determine whether the catheter should be rotated clockwise or counterclockwise, or whether it should be pushed or pulled. Moreover, the LP implantation was conducted under local anesthesia, and using 3D transesophageal echocardiography could cause additional discomfort for the patient and might require deep sedation or general anesthesia. Therefore, we decided not to use those modalities.

## Conclusion

The Aveir LP and delivery catheter system became caught on and reeled in intracardiac tissue when it was re-docked and rotated. If the protective sleeve does not advance smoothly, the Aveir system should be placed back in the tether mode and the position of the delivery catheter should be changed.

## Disclosures

All authors have no conflicts of interest to disclose.

## Appendix Supplementary Data

Supplemental Video 1 The leadless pacemaker was attempted to recapture the device and change its location due to the unacceptable pacing threshold. The protective sleeve did not advance beyond the middle of the device.

## Footnotes

Appendix Supplementary data associated with this article can be found in the online version at https://doi.org/10.1016/j.hrcr.2024.04.010.
